# A new satellite of manganese revealed by extended-range high-energy-resolution fluorescence detection

**DOI:** 10.1107/S1600577523002539

**Published:** 2023-04-07

**Authors:** Nicholas T. T. Tran, Daniel Sier, Tony Kirk, Chanh Q. Tran, J. Frederick W. Mosselmans, Sofia Diaz-Moreno, Christopher T. Chantler

**Affiliations:** a School of Physics, University of Melbourne, Melbourne, Victoria 3010, Australia; b Department of Chemistry and Physics, La Trobe University, Melbourne, Victoria 3086, Australia; c Diamond Light Source, Didcot, United Kingdom; University of Essex, United Kingdom

**Keywords:** XR-HERFD, HERFD, RIXS, manganese, synchrotron

## Abstract

The discovery of a new physical process in manganese metal is reported. This process will also be present for all manganese-containing materials in condensed matter. The process was discovered by applying our new technique of XR-HERFD (extended-range high-energy-resolution fluorescence detection), which was developed from the popular high-resolution RIXS (resonant inelastic X-ray scattering) and HERFD approaches. The acquired data are accurate to many hundreds of standard deviations beyond what is regarded as the criterion for ‘discovery’. Identification and characterization of many-body processes can shed light on the X-ray absorption fine-structure spectra and inform the scientist on how to interpret them, hence leading to the ability to measure the dynamical nanostructures which are observable using the XR-HERFD method. Although the many-body reduction factor has been used universally in X-ray absorption spectroscopy in analysis over the past 30 years (thousands of papers per year), this experimental result proves that many-body effects are not representable by any constant reduction factor parameter. This paradigm change will provide the foundation for many future studies and X-ray spectroscopy.

## Abstract 2

A new physical process in manganese, present for manganese-containing materials and materials science, has been discovered by applying our new technique – XR-HERFD (extended-range high-energy-resolution fluorescence detection) – developed from high-resolution RIXS (resonant inelastic X-ray scattering) and HERFD (high-energy-resolution fluorescence detection).

## 1. Development of RIXS and HERFD

Early experiments leading into the synchrotron era used resonant X-ray Raman spectroscopy (RXRS), revealing the basic idea of resonant decay with production of a characteristic X-ray spectrum (Sparks, 1974 ▸; Bannett & Freund, 1975 ▸; Eisenberger *et al.*, 1976*a* ▸,*b* ▸). Fluorescence scattering, investigating the X-ray absorption near-edge structure (XANES) and X-ray absorption fine structure (XAFS) of X-ray absorption spectroscopy (XAS), was developed by Jaklevic *et al.* from 1977 to 1993 (Jaklevic *et al.*, 1977 ▸; Jaklevic *et al.*, 1993 ▸) to become one of the most commonly used techniques at a synchrotron or for nano- and local structural determination. The combination of these two techniques provided the basis for a new technique: resonant inelastic X-ray scattering (RIXS) (Ament *et al.*, 2011 ▸; Gel’mukhanov *et al.*, 2021 ▸; Kang *et al.*, 2020 ▸; House *et al.*, 2021 ▸; Lee *et al.*, 2014 ▸; Lu *et al.*, 2021 ▸; Bisogni *et al.*, 2016 ▸). This is measured in the below-edge region. High-energy-resolution fluorescence detection (HERFD) (Glatzel *et al.*, 2005 ▸; Bauer, 2014 ▸; Hayama *et al.*, 2021 ▸; Friebel *et al.*, 2015 ▸; Agote-Arán *et al.*, 2019 ▸; Arias-Egido *et al.*, 2021 ▸; Lafuerza *et al.*, 2020 ▸) is measured in the full excitation and detection space and particularly in the above-edge region. Resonant *inelastic* X-ray scattering is a physical process, and ‘RIXS’ is a modern advanced technology for investigating such processes. Distinctions have been made between RIXS and resonant X-ray emission spectroscopy (RXES) on the basis of the measured data set axes, yet the physics and processes are identical. HERFD-XAS involves one-dimensional scans in the incident energy; and HERFD-XES (X-ray emission spectroscopy; usually called XES) involves one-dimensional scans in the detected emission energy. However, the theory and experiment should include the two-dimensional structure in both axes.

Authoritative and insightful reviews of the basic principles of RIXS and HERFD started early (Blume, 1985 ▸; Kotani & Shin, 2001 ▸). The realization of RIXS as a viable experimental technique commenced about 1991 (Hämäläinen *et al.*, 1991 ▸). This led to the development of technology directed towards purpose-built synchrotron beamlines and high-resolution detection. Early work highlighted the significance of understanding near-edge structure, geometry, symmetry and oxidation state (Etalaniemi *et al.*, 1992 ▸), and band structure and insights into solid state effects on characteristic spectra (Carlisle *et al.*, 1995 ▸). RIXS can discriminate between bound and continuum states; and its popularity has increased for recent studies that have investigated charge-transfer behaviour (Bisogni *et al.*, 2016 ▸), magnetic excitations in superconductors (Lee *et al.*, 2014 ▸), analysis of Mott insulators (Ivashko *et al.*, 2019 ▸), and more recently battery materials (House *et al.*, 2021 ▸). As a result, RIXS has great applicability for structural analysis needing meV resolutions, particular for fractional charge and optical waves. However, in the current investigation, we are looking for electronic states, transitions and structure above this energy regime.

Manganese, *Z* = 25, has hole widths of Γ_*K*_ = 1.11 eV; Γ_*L*III_ = 0.36 eV; Γ_*L*II_ = 0.97 eV; Γ_*L*I_ = 6.2 eV (Campbell & Papp, 2001 ▸). Hence a typical (*K*) hole-width in XAS or XES across the transition metals might yield a limiting resolution of 1–4 eV in the incident energy or emission energy axis, whereas the crystal analyser can be limited by the diffraction crystal energy width or by the incident energy bandwidth and optics. This can be, for example, 1–2 eV or 0.4–0.8 eV, and indeed even below 100 meV in recent RIXS pre-edge studies (Ament *et al.*, 2011 ▸) and for soft X-ray beamlines including for *L* pre-edge spectra (Kang *et al.*, 2020 ▸; Huotari *et al.*, 2008 ▸).

The two-dimensional incident–emission energy surface can yield amazing insight into structure, and into physical processes in the pre-edge and near-edge region. These have been the mainstay of this field. Major recent reviews have captured some of the excitement, notable theory, understanding and applications (Ament *et al.*, 2011 ▸; Gel’mukhanov *et al.*, 2021 ▸; Glatzel *et al.*, 2013 ▸). This is particularly the case for the key output of HERFD, which reaches below the lifetime (*K*) hole-width in spectral resolution, and RXES/RIXS. This provides detailed near-edge characterization of the pre-edge X-ray spectrum. RIXS has played a significant role in the study of complex systems such as magnon dispersion (Braicovich *et al.*, 2009 ▸), high-temperature superconductivity (Chen *et al.*, 2011 ▸; Nag *et al.*, 2020 ▸), catalysis (Timoshenko & Frenkel, 2019 ▸) and battery cathodic time-dependent hysteresis (House *et al.*, 2020 ▸).

Manganese systems have been studied at many important beamlines (Ament *et al.*, 2011 ▸; Kotani & Shin, 2001 ▸; De Groot, 2001 ▸), with RIXS line scans of MnO, oxides, MnF_2_ and for *K*β, and contour plots of the RIXS pre-edge plane of oxides (Glatzel *et al.*, 2004 ▸, 2013 ▸; Glatzel & Bergmann, 2005 ▸).

However, there are persisting challenges of theory for RIXS or HERFD in explaining Coster–Kronig and resonant Auger transition processes (De Groot, 1996 ▸, 2001 ▸). Limitations of the existing theory have been discussed (Bauer, 2014 ▸; Kotani & Shin, 2001 ▸; Gallo & Glatzel, 2014 ▸; Norman & Dreuw, 2018 ▸; Kroll *et al.*, 2021 ▸) including energy offsets, structure, resolution and magnitude of features. Even representing and then fitting the raw data presents key challenges, leading to the first soft X-ray and limited stack plots (Kotani & Shin, 2001 ▸; Jiménez-Mier *et al.*, 1999 ▸) as opposed to earlier line scans. The first 2D plots were analysed with line scan integrals (Glatzel *et al.*, 2004 ▸, 2013 ▸; Glatzel & Bergmann, 2005 ▸) ‘because producing the full 2D map is difficult’.

Developments of the theory of RIXS and HERFD have continued apace based around the Kramers–Heisenberg formula,

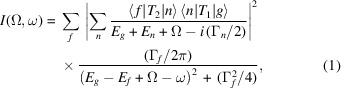

where |*f*〉, |*n*〉 and |*g*〉 are the final, intermediate and ground states, respectively. *T*_1,2_ are the transition operators between the ground and intermediate states and the intermediate and final states, respectively. Ω and ω are the incident and emission X-ray energies, respectively. Γ_*n*_ and Γ_*f*_ are the lifetime widths of the intermediate and final states, respectively. The modern experimental techniques per se have now been successful for decades. Developments of advanced beamlines have improved resolution, *e.g.* at the Cu *K*-edge, from 1.5 eV in 1999 to 100 meV in 2007, with a simultaneous flux-on-target improvement of order 10^3^; and for the Cu *L*-edge (on different beamlines) similar improvements have been made (Ament *et al.*, 2011 ▸).

These techniques are ideal for investigating near-edge geometry, multipole processes, oxidation states and Fermi levels of simple and complex materials. HERFD is often performed using high-energy-resolution Bragg diffraction analyser crystals with a curved crystal geometry, to focus the radiation in a point-to-point setup onto a (multi-element) pixel detector. Because Bragg diffraction with a focusing geometry is used with large crystal radii, the spectral resolution can be much better than energy-dispersive detection fluorescence scattering or energy-dispersive detection XES. HERFD requires scanning of the emission energy and separates elastic scattering, RIXS and non-resonant inelastic X-ray scattering (NRIXS) processes because of the higher resolution, and is usually a slower technique. RIXS measurements investigate pre-edge oxidation and density of states. HERFD-XAS measures near-edge, at the beginning of XANES and pre-edge features. The structural variation with oxidation state reveals many details. Typically, comparisons of line integrals or line scans with XAS theory or experiment have required experimental offsets and background subtraction. A challenge of any high-resolution measurement is that agreement between theory and experiment requires more careful measurement and calibration of experiment and of theoretical offsets, amplitudes and structure. Recent developments of HERFD have investigated *K*β transitions and satellites, and have led to the creation of the exciting field of valence-to-core spectroscopy in the last few years (Bauer, 2014 ▸). Satellites occur in the same or nearby spectral region. The current success of valence-to-core spectroscopy, targeting a well known dominant feature (*K*β), argues for a search for new structure and processes which directly affect current analysis and theoretical assumptions in the field.

We began this inquiry for two reasons: firstly, to search for new structure and science which could be revealed by the extraordinary resolution and structure of two-dimensional XES maps. While much theory is devoted to single-electron excitation and often electric dipole transitions, higher-order relativistic quantum mechanics is necessary for much of the advanced phenomena we observe today. The main 1*s* hole for a *K*-edge excitation and *n = 2* hole for *L*-edge excitation yields the ‘diagram spectrum’ of X-ray emission spectroscopy. Hence the characteristic (X-ray) *K*α spectrum is often seen as a 1*s*–2*p* electric dipole transition. However, many-body satellites occur whenever electron correlation occurs and especially in two-electron correlation and excitation. The term satellite is a general term in spectroscopy relating to any substructure of a dominant peak whether it be in the X-ray, visible, XPS or IR spectral regions. Sometimes this reveals little of the condensed matter system. Often there may be many-body effects of correlated systems, molecular orbital interactions *etc*., and theory may struggle to explain them. If satellites involve two-electron emission, they yield ‘satellite spectra’ where two holes are created, for example yielding a ‘1*s*3*d* satellite spectrum’ or 1*s*3*d*–2*p*3*d* transitions.

Many-body processes and satellite spectra are present in X-ray spectroscopy most of the time (Hölzer *et al.*, 1997 ▸). This is especially the case for two-electron processes, collective excitation and deexcitation processes and shake-up and shake-off processes, where double ionization is the consequence of excitation by photon impact above the ionization edge.

However, in most experiments, including reference standard experiments and spectra obtained at reference laboratories such as NIST (Mendenhall *et al.*, 2017 ▸), these satellite spectra can only be hinted at as slight asymmetries in the overall XES spectrum, and until recently neither experiment nor theory have been able to isolate or separate these (Pham *et al.*, 2016 ▸; Nguyen *et al.*, 2021 ▸). Indeed, the physical mechanism and quantum mechanics of such spectra are not yet fully understood and are subject to debate. An initial investigation of normal XAS spectra and their evolution of amplitude above the edge (Nguyen *et al.*, 2021 ▸) has been made, but the resolution has not been adequate to probe the details of discussion here and the influence of many-body processes on XAFS.

Secondly, we investigated this to initiate a search into the physical meaning and nature of the many-body reduction factor 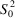 used extensively in XAFS, RIXS and HERFD-XAS fitting procedures for the last 40 years. The only way to measure a many-body term such as 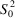 is to observe, isolate and measure the magnitude of specific dominant many-body processes, *i.e.* to look for and find the satellites.

**Figure 1:**
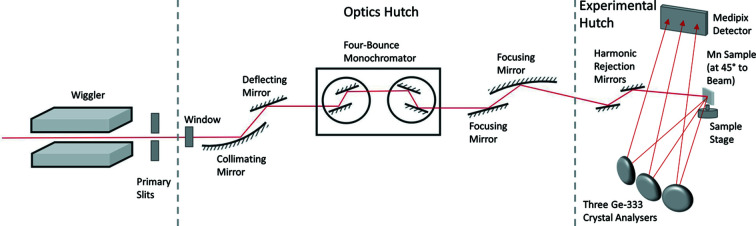
Experimental schematic. Three Ge-333 crystal analysers lie adjacent to each other on the Rowland circle and direct X-ray emissions to three separate regions on the Medipix detector. This novel set-up provides three independent measurements of the XR-HERFD and allows uncertainty determination (see Section S1 of the supporting information for more information).

Here we report the discovery of a satellite of manganese in manganese metal showing evolution with incident energy, and demonstrating definitively that the many-body reduction factor is energy-dependent. We use a new technique – extended-range high-energy-resolution fluorescence detection (XR-HERFD) – to observe this process. This technique has evolved from experience with RIXS and HERFD experiments. We used the Diamond I20 beamline, scanning branch (Diaz-Moreno *et al.*, 2018 ▸) (Fig. 1 ▸; see the supporting information).

**Figure 2:**
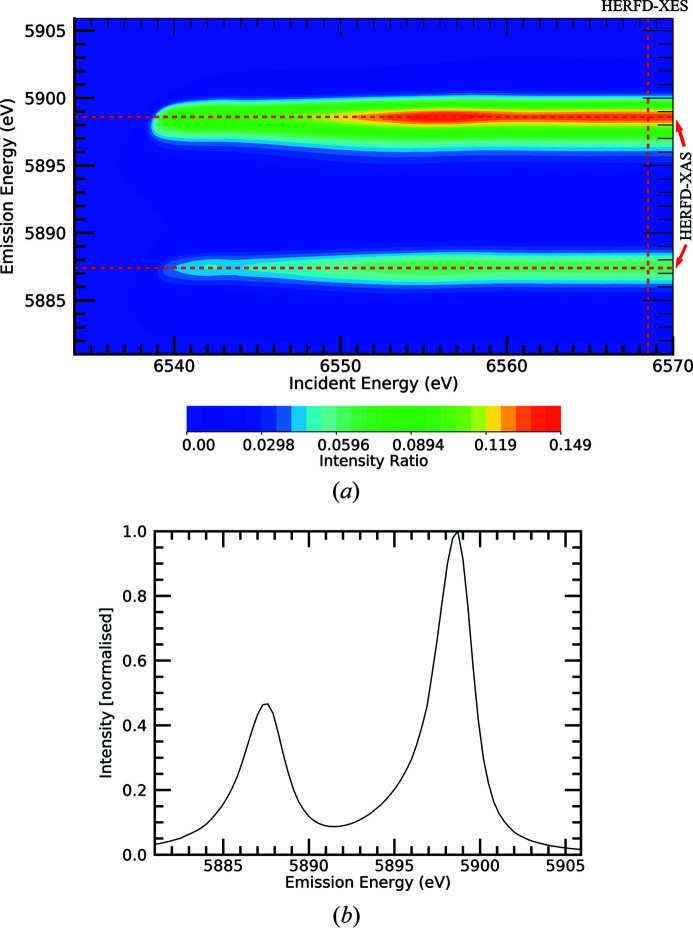
(*a*) HERFD contour plot of the ratio FF/I1 of the average fluorescent count, FF, to upstream ion chamber count, I1, of the crystal analysers for the Mn metal foil, versus incident energy and emission energy. The horizontal line at 5898.6 eV emission energy is a HERFD-XAS scan at the peak of the *K*α_1_ signal; the horizontal line at 5887.4 eV emission energy is a HERFD-XAS scan at the peak of the *K*α_2_ signal (Nguyen *et al.*, 2021 ▸); the potential RIXS pre-edge resonance signal is weak for Mn metal and restricted to below an incident energy of 6540 eV. The vertical line is a HERFD-XES characteristic spectrum. (*b*) Integration of the ratio of fluorescent counts to upstream ion chamber counts for Mn foil at incident energies from 6568.50 eV to 6570 eV, normalized to the maximum value at the *K*α_1_ peak. The maximum integrated fluorescent to upstream ion chamber count ratio is 0.819 at the *K*α_1_ peak.

Fig. 2 ▸(*a*) shows an example of a full RIXS/HERFD map of the manganese metal foil. The detailed structure and development of intensity above the *K*-edge, for a single line scan or pixel scan, would be a horizontal line at the peak HERFD-XAS (*K*α_1_) at 5899 eV, or HERFD-XAS (*K*α_2_) at 5888 eV. The onset of the characteristic *K*α spectrum for any line scan along the vertical axis corresponds to HERFD-XES and reveals the XES spectrum. In the literature, this is often integrated to yield a characteristic profile. Since this plot is for Mn metal foil, no pre-edge resonant features are observed in the spectrum, because the bound–bound features are occupied below the Fermi level. Hence the RIXS pre-edge region is absent or featureless. From such HERFD measurements we can look for a relatively stable *K*α XES spectrum by integrating the ratio of fluorescence intensity to ion chamber intensity, FF/I1, as a function of incident energy for the last few vertical XES slices of a typical RIXS or HERFD scan [in this case 6568.50 eV to 6570 eV; Fig. 2 ▸(*b*)].

Fig. 2 ▸(*b*) shows the weighted mean of the ratio of fluorescent counts to upstream ion chamber counts of the crystal analysers for multiple repeated scans of Mn foil. This has been integrated over incident energy from the RIXS measurements from 6568.5 eV to 6570.0 eV (six scans). This demonstrates the well known characteristic spectra asymmetry and hence structure. Whereas HERFD-XAS is high resolution and the RIXS pre-edge is high resolution, this XES spectrum [Fig. 2 ▸(*b*)] is limited by the typical broadening along this axis of the *K*-edge hole-width of order 1.1 eV. This spectrum is comparable with the best in the literature and displays structure. However, as has been recorded in all the past literature on RIXS or HERFD, we do not see separate or identifiable satellite structure indicative of new physical processes.

Therefore, we investigate the wider XES spectrum, *i.e.* in the emission energy axis towards 5940 eV, and the noise level. We quantified uncertainty and statistic as the standard error σ_se_ on the same scale, which is the uncertainty estimate of the weighted mean, from the repeated and sequential scans. This is an important development and requirement to look for significance and anomalies in the spectrum (see Fig. S1 of the supporting information). This did not discover a new physical process. By presenting this on a logarithmic scale we investigated the spectral detail further (see Fig. S2). Given the excellent statistics of the synchrotron beamline and detection system, the spectra obtained are very clear and reached the noise level, yet again no well resolved new structure was observed.

## 2. Extended-range investigation in two-dimensional XES space

**Figure 3:**
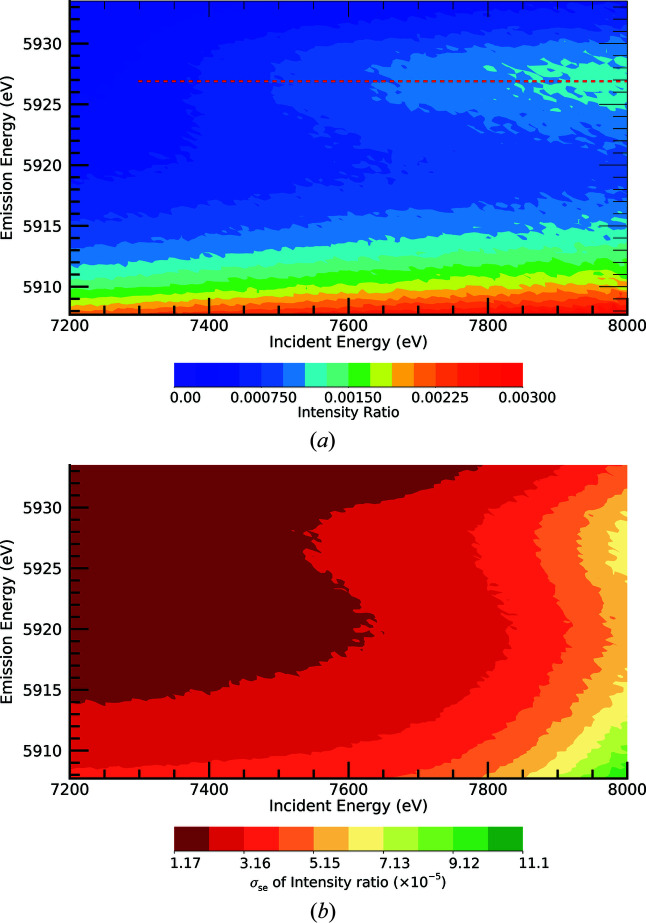
(*a*) XR-HERFD contour plot of Mn in the key energy regions of interest showing the ratio of fluorescent counts to upstream ion chamber counts. The tail of the *K*α_1_ peak can be seen at low fluorescent energies. A new satellite is discovered, marked by the dashed red line at the emission or fluorescence energy of 5927 eV. It appears to begin from ∼7400 eV in the incident energy. (*b*) Propagated standard error σ_se_ contour plot for the XR-HERFD map of the crystal analyser fluorescent to upstream ion chamber count ratios in (*a*).

We then explored a novel idea to investigate particular regions of the two-dimensional XES plane far away from conventional investigations. We investigated the incident energy range from 7200 eV to 8000 eV, well above the typical range for RIXS of 6530 eV to 6570 eV (for Mn) – which is typically up to 30 eV above the edge – and the emission energy from the normal onset 5880 eV (for Mn) but now up to the novel 5934 eV of the previous spectra. We present the contour plot of this extended plane [Fig. 3 ▸(*a*)]. A new feature appears.

This represents a physical process which has not been observed in manganese spectra. This feature is clearly not a constant and instead evolves as the incident energy increases above 7400 eV. It also lies well in the tail of the Mn *K*α characteristic spectrum, and could not be seen by RIXS or HERFD-XAS or HERFD-XES scans – RIXS/RXES is by definition resonant, and to see these new physical processes one must look far beyond the resonant region. However, it can be seen by collecting the emission from the sample in non-resonant conditions, well above the absorption edge, as a function of the incident energy. This type of investigation is then called XR-HERFD (eXtended Range HERFD). Because of our multiple energy scans and our separated crystal analyser foci, we can present contour plots for standard error uncertainties σ_se_ for data presented in Fig. 3 ▸(*a*) as Fig. 3 ▸(*b*).

**Figure 4:**
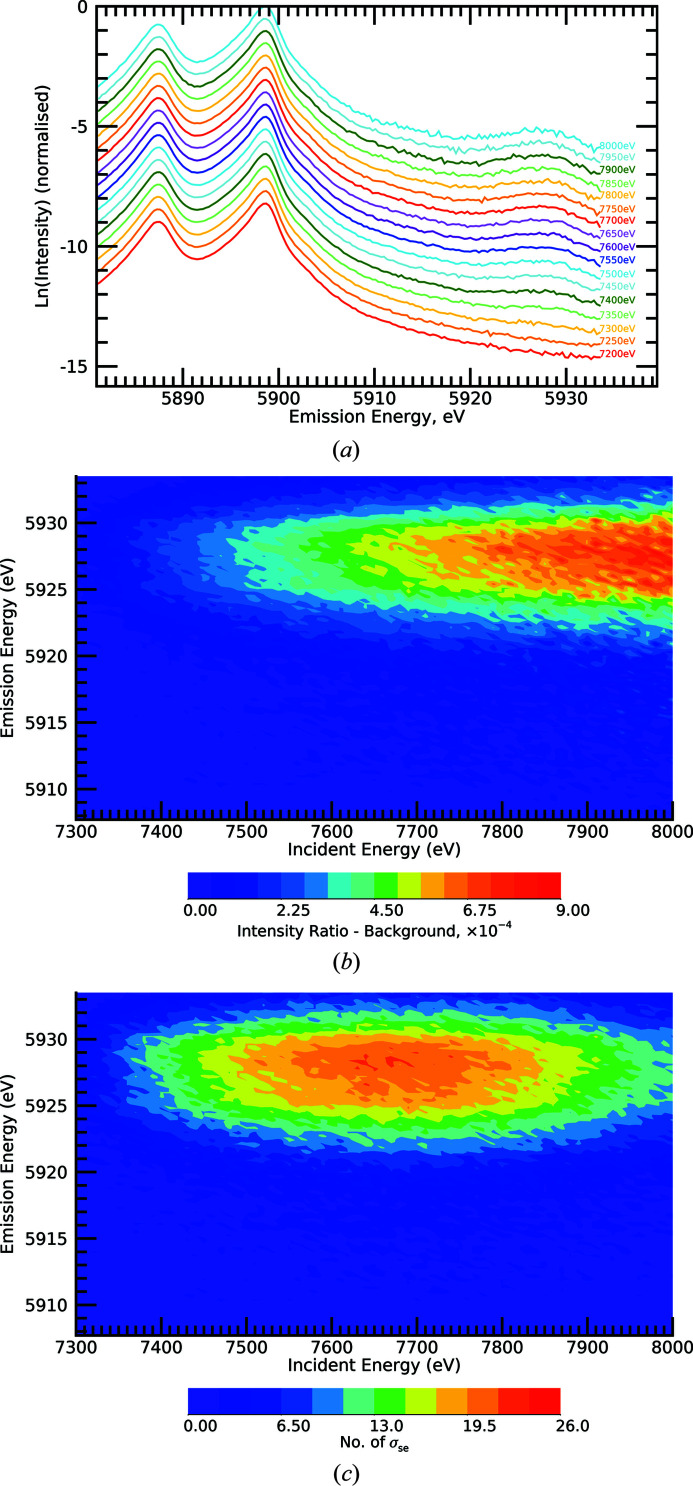
(*a*) Stack plot of HERFD-XES slices of the manganese metal XR-HERFD data at labelled intervals of 50 eV in incident energy. Evolution of the new satellite with increasing incident energy is observed. The onset of the satellite is below 7300 eV. (*b*) XR-HERFD map after background subtraction. (*c*) XR-HERFD contour plot of the significance of the satellite region. This shows the number of standard errors of the satellite after a background subtraction of the data. At peak regions on this plot, along the peak of the new process, each data point in the two-dimensional area of the new peak has a significance of more than ten standard errors σ_se_. This corresponds to a total error signature that is many hundreds of standard errors. Conventionally, a discrepancy of three to ten standard errors is expected for a discovery. This is indicative of a new satellite.

Stack plots dramatically display the evolution of the satellite from slices of the XES plane on a log scale [Fig. 4 ▸(*a*)]. The satellite is clear to see – the noise level for an isolated scan is moderately clear by eye, the onset is quite strongly observed (at least down to 7300 eV) and evolves clearly as the incident energy increases. We can investigate the hypothesized new structure by performing a background subtraction of the *K*α dominant peak. Fig. 4 ▸(*b*) presents this background subtraction and the new process appears strong and significant.

Fig. 4 ▸(*c*) shows the number of standard errors σ_se_ for each point of the previous map for the satellite data. The significance is greater than ten standard errors at each data point in the region of the satellite peak. This lies far above the level of discovery for each data point in the two-dimensional region along the area of the new process, for a total of many hundreds of standard error observations. We note that the normalized signal continues to increase but (see Section S1 of the supporting information) the system approached the monochromator mirror cut-off, so that the counts (incident and fluorescent) began to decline and ergo the significance per point is reduced at the highest energies.

## 3. The newly observed process

This new peak is an *n* = 2 satellite spectrum and a shake-off many-body process. It may be the 2*p* satellite spectrum to the diagram 1*s*–2*p* fluorescence spectrum. (A satellite is a double ionization event with the main 1*s* hole and a second, satellite, 2*p* hole so that two electrons are knocked out of the atom in the same process.) If so, it represents a transition from the hole state Mn 1*s* ^−1^2*p* ^−1^ to 2*p* ^−2^, or 1*s*2*s* ^2^2*p* ^5^3*s* ^2^3*p* ^6^3*d* ^5^4*s* ^2^ to 1*s* ^2^2*s* ^2^2*p* ^4^3*s* ^2^3*p* ^6^3*d* ^5^4*s* ^2^. Because the dynamics of the transition are complex, other possibilities may exist. These data show the evolution from the onset of the transition strength with incident energy. We have used one-electron relativistic quantum mechanics to predict an onset energy for atomic manganese of ∼7200 eV to 7300 eV. To investigate such processes, we have predicted:

(1) The incident, excitation onset energy when the channel or process becomes available (which is roughly the edge energy plus the satellite electron binding energy, neglecting wavefunction recoupling).

(2) The fluorescence energy or XES energy when the process appears relative to the peak of the characteristic spectrum [this is more challenging theoretically but may be estimated from the best-tested spectra of copper, where a cognate feature has been observed (Mendenhall *et al.*, 2017 ▸)]. As predicted, we see it here to be about 30 eV above the Mn *K*α_1_ peak energy.

(3) The range over which the structure and onset occurs. This can be keV in XAS space, and about 10 eV in XES space as predicted and seen in Fig. 3 ▸.

Theoretical predictions of the existence of this feature depend upon a large range of approaches and assumptions including: the relativistic approach, the nature of the configuration state expansion and basis set, and the methodology for incorporating relativistic and higher-order effects. With our advanced quantum mechanical multi-configuration analysis (MCDHF; Pham *et al.*, 2016 ▸; Nguyen *et al.*, 2021 ▸), more information will be revealed. The data are important for atomic and molecular science because the type of process and the nature of the process have not been seen nor investigated before, yet affect both atomic theory and the question of solid state science and bonding. In X-ray science and synchrotron science, it is especially important because it represents a many-body transition.

Many-body transitions are the bane, or major challenge, to current advanced theory and modelling of XAFS and related technologies such as RIXS and HERFD, especially because these are difficult to compute in any density functional theory (DFT) including time-dependent DFT. A major reason for this theoretical challenge is electron correlation, which for complex open-shell atomic or molecular or solid-state theory are significant open questions, all of which have ill-defined assumptions and approaches. Indeed, this remains true even for purely atomic relativistic theory. In this experiment we have been able to observe and measure them for the first time, including for interactions far from the high-energy impact approximation limit.

XAFS, whether for transmission or fluorescence experiments, or even high-resolution HERFD-XAS spectra, is currently modelled with a key (fitting) parameter 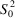 , the ‘many-body reduction factor’, which is stated to represent the loss of correlation and signal of the quantum interference of the photoelectron wave due to many-body processes – exactly as we observe explicitly in this experiment.

Indeed, there are major challenges which we will not go into here. However, 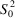 is usually fixed to a convenient value, *e.g.* 0.9, 0.8 or something else in sympathy with naïve estimates; or empirically fitted over the energy or wavevector spectral range of the photoelectron quantum interference in energy or wavevector *k*-range. This parameter, as a fitted or fixed variable, is totally correlated with the coordination number, so if we do not know 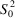 then the uncertainty in determining the coordination number increases. Understanding or defining this removes significant correlation uncertainty in the coordination number.

Our data and results prove that 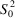 can in fact now be directly investigated. And we have observed that it has an energy dependence. We have proven that 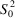 is energy- and wavevector-dependent. The results show that 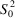 depends upon the integration range and resolution of the optics and detector system; and it should decrease with increasing energy. Hence, for example, this demonstrates that the value of 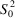 in an XAS or fluorescence XAS experiment will be different with a different *k*-range of observation or fitting, and will be different from the corresponding HERFD-XAS experiment. In modern EXAFS theory the fine structure is often modelled using the equation

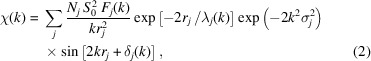

where *N*_*j*_ is the degeneracy of the path, *F*_*j*_(*k*) is the back-scattering amplitude, *r*_*j*_ = (1 + α)*r*_0*j*_ is the adjusted half-path length, (1 + α) is the thermal expansion coefficient, *r*_0*j*_ is the input half path length, δ_*j*_(*k*) is the phase shift, σ_*j*_ is the Debye–Waller factor which accounts for thermal movements and is defined as the mean square variation of the scattering path length *j*, and λ_*j*_(*k*) is the inelastic mean free path function of the photoelectron. 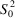 corresponds to many-body effects and is usually fitted or defined as a constant. It may be written as

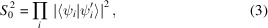

where the unprimed wavefunctions relate to the unperturbed atom and the primed wavefunctions to the atom with core hole(s) present (Rehr *et al.*, 1978 ▸).

In this work we see explicitly the change, *i.e.* the reduction of 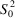 , as one moves above the onset energy of 7200 eV, especially for normal XAS studies in absorption or fluorescence, whether using regions of interest or a full-spectrum analysis. This observation would apply to all manganese-containing materials. Qualitatively 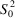 would decrease as one moves in incident energy across the range from 7100 eV to 8000 eV and above.

Shake-off processes are forbidden when the incoming photon has an energy below that of the sum of the binding energies of the core and outer electron. Hence 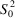 must change with energy and when more excitation and ionization channels become available. The same is true of shake-up processes; however, their discrete nature makes any change in 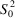 brief and negligible. XR-HERFD is the perfect tool for investigating these phenomena, as it enables us to observe at exactly what energy shake-off processes occur and their relative magnitude.

Many more of our studies of XR-HERFD will reveal the detailed structure and provide a logical framework for future theory and experimental insights for all the applications where X-ray science, XAFS and HERFD-related experiments are important. The hypothesis that this work can be extended to compounds and materials of manganese-containing systems can be investigated, including how the changing quantum system affects the detailed spectra.

Future explorations of XR-HERFD will now be able to explore a wide range of fields and fundamental and advanced questions including: the structure of these new satellites; innovative theory required to predict them; finding which many-body processes are dominant for which energy ranges and for which materials; establishing additional satellites and other many-body processes that are normally hidden from high-resolution X-ray spectroscopies; and investigating how the many-body processes vary with existing models and experimental data for molecular bonding, band theory and crystal fields. All of the above have consequences for current theoretical and experimental analysis of nanostructure, dynamic and nano-structure revealed by such studies, and other as yet unknown opportunities.

## 4. Related literature

The following references, not cited in the main body of the paper, have been cited in the supporting information: Plackett *et al.* (2013 ▸); Diaz-Moreno *et al.* (2009 ▸)

## Supplementary Material

Section S1: Methods. Figures S1 and S2. DOI: 10.1107/S1600577523002539/rv5171sup1.pdf
