# Supplementary material for: A new satellite of manganese revealed by extended-range high-energy-resolution fluorescence detection
Source: J Synchrotron Radiat. 2023 Apr 7;30(Pt 3):605–12. doi: 10.1107/S1600577523002539 (PMC10161895; doi:10.1107/S1600577523002539)
Supplement: Supplementary file 1 [file s-30-00605-sup1.pdf]

JOURNAL OF SYNCHROTRON RADIATION

Volume 30 (2023)

Supporting information for article:

A New Satellite of Manganese revealed by Extended-Range High-Energy-Resolution Fluorescence Detection

Nicholas T. T. Tran, Daniel Sier, Tony Kirk, Chanh Q. Tran, J. Frederick W. Mosselmans, Sofia Diaz-Moreno and Christopher T. Chantler

S1. Methods

The experiment was conducted at Diamond Light Source on the I20-Scanning beam line (Diaz-Moreno *et al.*, 2018; Diaz-Moreno *et al.*, 2009) at room temperature for a sample of manganese foil. The 4-bounce monochromator consists of two synchronised counter rotating axes which have a double crystal configuration which together are set in a dispersive configuration (Diaz-Moreno *et al.*, 2018). The monochromator uses Si(111) crystals which allows selection of energies between 4 and 20 keV (Diaz-Moreno *et al.*, 2018). Harmonic rejection mirrors remove harmonics if the beam energy is less than 18 keV. The Harmonic Rejection Mirror cutoff is 11.5 keV. The third experimental slit is followed by an upstream monitor, shutter and upstream ion chambers which measure the incident photon counts before the sample. The fwhm beam size on the sample was 400 μm × 300 μm.

The spectrometer follows Hayama *et al*. (Hayama *et al.*, 2021) but with a novel experimental set up of the crystal analysers. Three Ge(333) analyser crystals of 100 mm diameter, masked by a 25 mm wide ring mask so only the central portion was visible, are aligned adjacent to each other on a Rowland circle of diameter 1m in a Johann configuration, focussing the X-rays to the detector, also on the Rowland circle. Normally all analyser crystals focus the emission on the same region of the Medipix detector. Importantly in this experiment, each analyser crystal is angled to focus the X-ray emissions from the sample to three distinct separate regions on the detector, giving three independent measurements of the HERFD plane and RXES, and providing an independent measure and determination uncertainty. We used a four element medipix detector developed at Diamond Light Source (Plackett *et al.*, 2013) with a detector size spanning 256 pixels by 1033 pixels, which corresponds to a size of 14 mm × 56 mm.

The 25 μm Mn foil sample is mounted on the sample stage at an angle of 45° to the beam. The manganese metal foil is coated on one side with a permanent polyester support, 0.125 mm, with the metal thickness 25μm (± 15%). The purity is specified as 98.7% by the suppliers, with typical impurities dominated by Pb 1.25%, and also Al 40, Ca 100, Cr 4, Cu 8, Fe 40, Mg 100, Si 50, parts per million. Notice that in XR-HERFD, RIXS and HERFD, none of these overlap with the detailed 2D spectral features observed in high-resolution. We orient the foil so that the metal side faces both the incident beam and the fluorescent detectors. These foils are quite stable in the strong beam and to general oxidation. We do not notice any rolling defects or pattern.

RXES measurements were taken of the Mn sample near the K edge as well as in a new range much higher in incident energy to investigate the presence of off axis contributions. The former scanned through incident energies from 6534.0 eV to 6570.0 eV in step sizes of 0.3 eV, and emission energies from 5881.0 eV to 5906.0 eV in step sizes of 0.3 eV. The second type of measurement scanned through incident energies from 7200 eV to 8000 eV with step sizes of 10 eV, and emission energies from 5881.0 eV to 5933.5 eV with a step size of 0.3 eV. Compared with the first type of scan, this

looks at a higher incident energy above the absorption edge, but also includes a larger range beyond the $K\alpha_1$ emission peak.

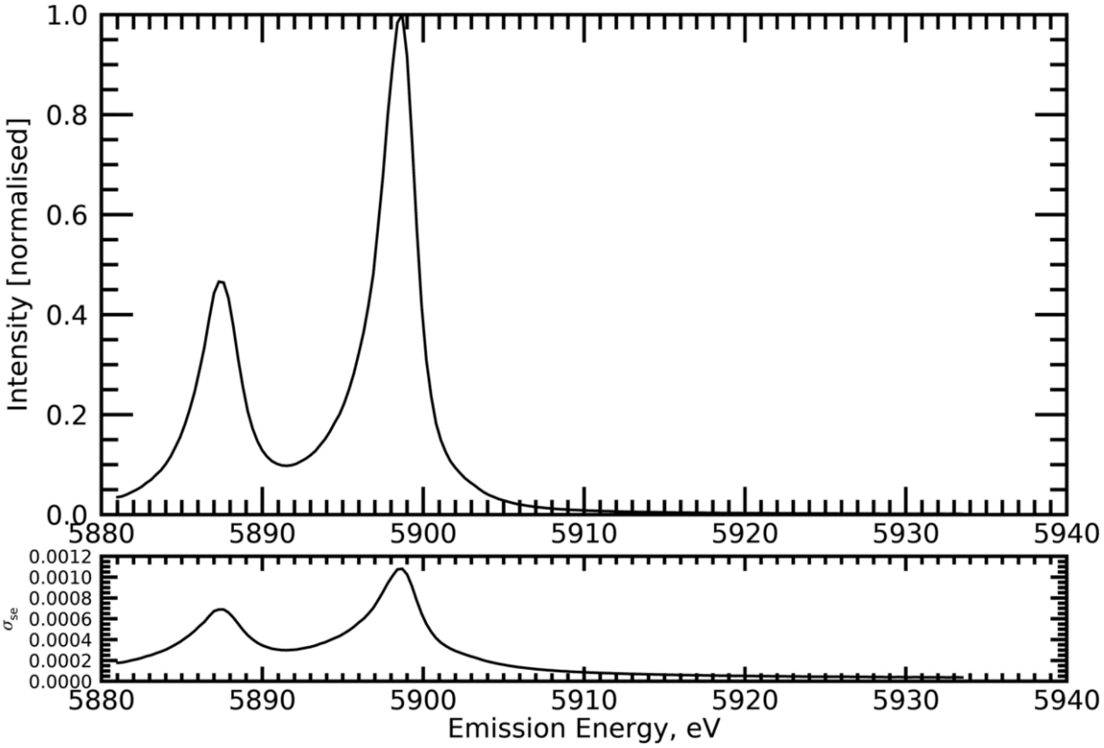

Figure S1 Integration of incident energies from 7200 eV to 7240 eV here (5 scans) compared with Fig. 1b which was from 6568.50 eV to 6570 eV (6 scans). The maximum integrated fluorescent to upstream ion chamber count ratio is 0.806 at the $K\alpha_1$ peak. The lower plot presents the standard error σ_{se}, the uncertainty estimate of the weighted mean.

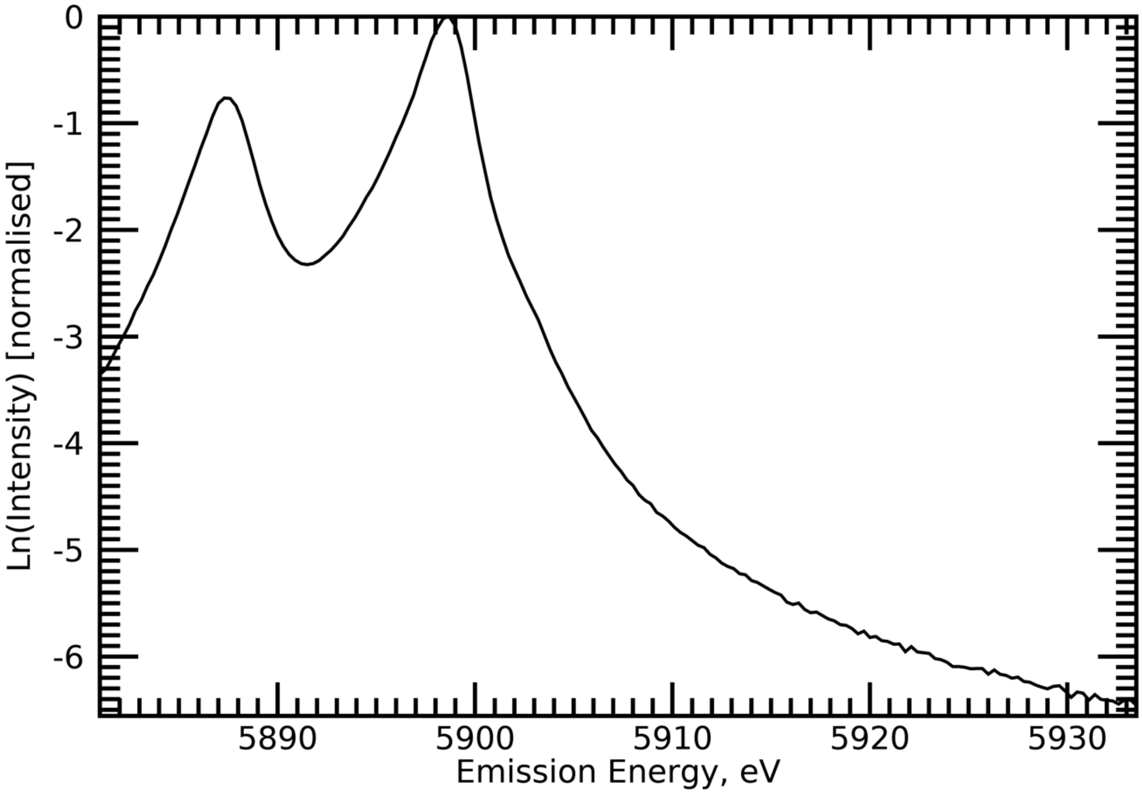

Figure S2 Natural log plots of integrated fluorescent to upstream count ratio of data in Fig. S1. The presence of a new physical process is not observable in this energy region (7200eV to 7240eV).
